# Single-fraction radiosurgery outcomes for large vestibular schwannomas in the upfront or post-surgical setting: a systematic review and International Stereotactic Radiosurgery Society (ISRS) Practice Guidelines

**DOI:** 10.1007/s11060-023-04455-8

**Published:** 2023-10-16

**Authors:** Constantin Tuleasca, Rupesh Kotecha, Arjun Sahgal, Antonio de Salles, Laura Fariselli, Ian Paddick, Bruce E. Pollock, Jean Régis, Jason Sheehan, John H. Suh, Shoji Yomo, Marc Levivier

**Affiliations:** 1 Neurosurgery Service and Gamma Knife Center, Centre Hospitalier Universitaire Vaudois (CHUV), Rue du Bugnon 44-46, BH-08, CH-1011 Lausanne, Switzerland; 2 Faculty of Biology and Medicine (FBM), University of Lausanne (UNIL), Lausanne, Switzerland; 3 Ecole Polytechnique Fédérale de Lausanne (EPFL, LTS-5), Lausanne, Switzerland; 4 Department of Radiation Oncology, Miami Cancer Institute, Baptist Health South Florida, Miami, FL USA; 5 Department of Neurosurgery, Unit of Radiotherapy, Fondazione IRCCS Istituto Neurologico C Besta, Milan, Italy; 6 Medical Physics Ltd, Queen Square Radiosurgery Centre, London, UK; 7 University of California Los Angeles, USA, NeuroSapiens and Rede D’Or São Luiz, São Paulo, Brazil; 8 Department of Radiation Oncology, Taussig Cancer Institute, Cleveland Clinic, Cleveland, OH USA; 9 Department of Neurosurgery, University of Virginia Health System, Charlottesville, VA USA; 10 Division of Radiation Oncology, Aizawa Comprehensive Cancer Center, Aizawa Hospital, Nagano, Japan; 11 Department of Functional and Stereotactic Neurosurgery, Assistance Publique-Hôpitaux de Marseille, Timone Hospital, Marseille, France; 12 Department of Neurosurgery, Mayo Clinic, Rochester, MN USA; 13 Department of Radiation Oncology, Sunnybrook Health Sciences Centre, University of Toronto, Toronto, Canada; 14 Rose Ella Burkhardt Brain Tumor and Neuro-Oncology Center, Neurological Institute, Cleveland Clinic, Cleveland, OH USA; 15 Institut Neurosciences des Systèmes, Aix-Marseille University, Institut National De La Santé Et De La Recherche Médicale, Marseille, France

**Keywords:** Vestibular schwannoma, Large, Radiosurgery, Gamma Knife, Hearing, Facial

## Abstract

**Purpose:**

To perform a systematic review of literature specific to single-fraction stereotactic radiosurgery (SRS) for large vestibular schwannomas (VS), maximum diameter ≥ 2.5 cm and/or classified as Koos Grade IV, and to present consensus recommendations on behalf of the International Stereotactic Radiosurgery Society (ISRS).

**Methods:**

The Medline and Embase databases were used to apply the Preferred Reporting Items for Systematic Reviews and Meta-Analyses (PRISMA) approach. We considered eligible prospective and retrospective studies, written in the English language, reporting treatment outcomes for large VS; SRS for large post-operative tumors were analyzed in aggregate and separately.

**Results:**

19 of the 229 studies initially identified met the final inclusion criteria. Overall crude rate of tumor control was 89% (93.7% with no prior surgery vs 87.7% with prior surgery). Rates of salvage microsurgical resection, need for shunt, and additional SRS in all series versus those with no prior surgery were 9.6% vs 3.3%, 4.7% vs 6.4% and 1% vs 0.9%, respectively. Rates of facial palsy and hearing preservation in all series versus those with no prior surgery were 1.3% vs 3.4% and 34.2% vs 40.4%, respectively.

**Conclusions:**

Upfront SRS resulted in high rates of tumor control with acceptable rates of facial palsy and hearing preservation as compared to the results in those series including patients with prior surgery (level C evidence). Therefore, although large VS are considered classic indication for microsurgical resection, upfront SRS can be considered in selected patients and we recommend a prescribed marginal dose from 11 to 13 Gy (level C evidence).

## Introduction

Stereotactic radiosurgery (SRS) is a widely accepted treatment for small to medium sized vestibular schwannomas (Koos grades I, II and III) [1, 2]. However, for large vestibular schwannoma (VS), using a definitional threshold of ≥ 2.5 cm or Koos Grade IV designation, most recommend microsurgical resection [3]. In particular, surgery should be considered when there are signs and symptoms of mass effect related to brainstem compression, cranial nerve (CN) neuropathy (other than CN VIII and particularly CN V), and/or presence of hydrocephalus [4]. Importantly, a wait and scan strategy is usually not recommended due to the possibility of life-threatening complications associated with tumor progression [5].

For a patient with a large VS who is not an optimal candidate for microsurgical resection, some type of fractionated radiation therapy is typically recommended. However, several centers have treated these patients with single fraction SRS, as they would do for smaller VS [6, 7]. Concerns of single-fraction SRS in these patients range from development of serious adverse radiation events (ARE), transient-tumor-expansion (TTE, also referred to as pseudoprogression), delayed time-to-response for patients who have symptomatic hydrocephalus, and late treatment failure necessitating surgery which may put the patient at an increased risk of surgical complications [8]. To date, there has yet to be a critical review of the published literature specific to this population to define efficacy and toxicity of this approach. Therefore, the purpose of this systematic review is to summarize the current literature specific to single fraction SRS for large VS, and provide treatment recommendations on behalf of the International Stereotactic Radiosurgery Society (ISRS) Guidelines Committee.

## Methods

### Systematic review

A systematic review of the literature was performed using the Preferred Reporting Items for Systematic Reviews and Meta-Analyses (PRISMA) approach [9].

### Search strategy

A search strategy evaluated the Medline and Embase databases to search for articles published from 1968 to June 2022. The following MESH terms or combination of those were used either in title/abstract: “radiosurgery” AND “vestibular schwannomas” AND “large” OR “Koos IV.”

### Inclusion criteria

We included prospective and retrospective studies, written in the English language, reporting patients treated for a large VS with either upfront single fractions SRS or those treated with single fraction SRS following surgery to a residual or recurrent tumor. We abstracted data for large VS based on those tumors with a maximum diameter ≥ 2.5 cm and/or classified as Koos Grade IV (large tumors with brainstem and cranial nerve displacement) [10].

**Fig. 1:**
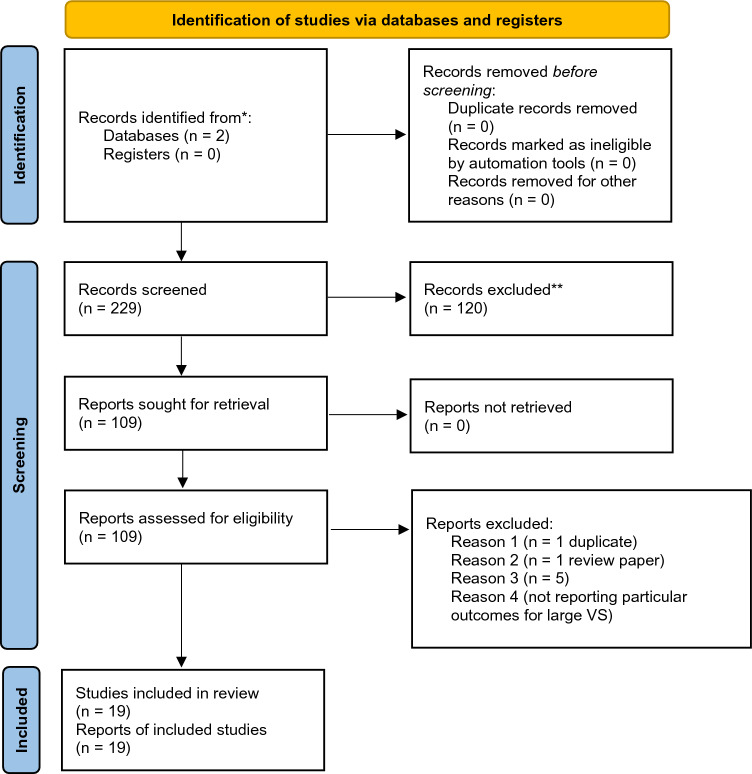
PRISMA flow diagram illustrating the study selection

We initially identified 229 studies, of which after screening abstracts, 120 were excluded (Fig. 1). The remaining 109 studies were further screened with a detailed review of the published manuscript. We retained only those 19 articles that met our strict inclusion and exclusion criteria[6, 8, 11–26]. 6/19 reported outcomes in patients who had not been previously resected[11, 15, 19, 23, 27, 28], and the remaining 13/19 studies included patients with prior surgical resection[6, 8, 12–14, 16–18, 21, 22, 25, 26]. Of note, the multicenter study by Pikis et al.[28] was kept in our analysis given the large number of cases and updated patient outcome information, despite potential overlap with single institution studies from those centers participating in the multicenter cohort. Demographic data are summarized in Table 1, and Table 2 summarizes dosimetric statistics and target volumes.

**Table 1:** Basic demographic data

	Year	Criteria	Patients (tumors)	NF 2	Prior microsurgery	Follow-up (months) Mean (median; range)	Male: Female	Age (years) Mean (median; range)
Series including patients with prior surgery								
Inoue et al	2005	> 3 cm	18 (20)	2/20	11/20	> 72	6:12	33–81
Chung et al	2010	> 3 cm	21	0/21	14/21	66 (53; 12–155)	9:12	–
Yang et al	2011	> 3cm median 9 mL	65	0/65	17/65	− (36; 1–146)	37:28	51 (19–89)
Zeiler et al	2012	3–4 cm	28	2/28	12/28	34.5 (-; 6–99)	13:12	− (56; 26–85)
Williams et al	2013	> 3 cm	24	0/24	9/24	− (48.5;7–211)	16:8	61.5 (62; 32–87)
Iorio-Morin et al	2016	> 4 mL	68	0/68	13/68	− (47; 6–125)	1.4:1	− (58; 16–85)
Lefranc et al	2018	Koos IV	86	0/86	14/86	74.4 (-; 36–192)	0.4:0.6	54.6 (23–84)
Huang et al	2018	> 3 cm & > 10 Ml	35	0/35	9/35	− (48; 6–156)	20:15	49.7 (-; 21–74)
Watanabe et al	2019	> 8 cc	19	0/19	9/19	− (98; 49–204)	8:11	− (71;29–91)
Stastna et al	2020	> 4 mL	73	0/73	4/73	− (66; 25.2–177.6)	29:44	− (61;23–84)
Mezey et al	2020	> 10 mL	103	0/103	18/103	74.4(-; 1.2–226.8)	46:57	61.5(-;20–88)
Hasegawa et al	2021	Koos IV	203	0/203	153/203	− (152; 12–277)	86:117	− (58;13–83)
Umekawa et al	2022	Koos IV	50	0/50	22/50	63(63;24–178)	–	57(-;28–86)
Series excluding patients with prior surgery								
Van de Langenberg	2011	> 6 mL, BS indent	33	0/33	0/33	− (30; 12–72)	15:18	54.8 (30–83)
Milligan et al	2012	> 2.5 cm	22	0/22	0/22	− (66; 26–121)	13:9	61 ± 15
Bailo et al	2016	> 25 mm	59	0/59	0/59	74.9 (79;36–164)	21:38	63.8 (-; 24–85)
Huo et al	2020	> 3.5 mL	19	0/19	0/19	28.7	10:9	57(-;38–73)
Ogino et al	2020	> 5 mL	170	0/170	0/170	− (61.2; 4.8–296.4)	93:77	− (61;21.1–39)
Pikis et al	2022	median 8.7 mL	627	0/627	0/627	Median 36	283:344	Median 54 (-; –-)

**Table 2:** Dose, target volume, local control and further surgical, shunt and/or SRS intervention

	Dose (Gy) Mean (median; range)	TV mean (median; range)	Radiological: Overall control; stable volume; decrease	Further surgery, SRS or shunt (time point if given)
Series including patients with prior surgery				
Inoue et al	10 (4/20); 11 (2/20); 12 (14/20)	15.2 (-; 5.3–28.5)	14/15; 6/15; 8/15	Resection: 1/15 resection (2 years after)
Chung et al	11.9 (12; 11–14)	17.3 (17.1; 12.7–25.2)	18/21; 17/21; 1/21	Resection: 2/21 (8 and 72 months after) Shunt: 2/21 (7, 17 months after) Omaya: 1/21 (11 months after)
Yang et al	− (12;11–15)	9 (-;5–22)	58/65; 18/65; 38/65	Resection: 7/65 (2 at 6 months and 5 at 2.5 years) Shunt: 3/65
Zeiler et al	12.5 (-;12–13)	9.7 (-; 6.9–10.6)	23/25; 9/25; 14/25	Resection: 1/25 Shunt: 3/25 (5, 11 and 24 months after)
Williams et al	− (11; 8–20)	− (9.5; 3.1–24.7)	16/18; 6/18; 10/18	Resection: 3/24 (at a mean of 46 months (6–102) after) SRS: 3/24 Shunt: 2/24
Iorio-Morin et al	12 (-;11–13)	-(7.4; 4–19)	64/68	Resection: 3/68 (4, 13, 36 months after) Shunt: 3/68
Lefranc et al	11(-; 10–12)	4.46 (-; 1.38–8.69)	78/86; 26/86; 60/86	Resection: 7/86 Shunt: 1/86
Huang et al	− (11;10–12)	− (14.8;10.3–24.5)	30/35; 5/35; 25/35	Resection: 5/35 (9 months-6 years after) Cysto-peritoneal shunt: 2/35
Watanabe et al	− (12, 10–12) minimum − (12;10–12)	− (11.5; 8–30.6)	15/19; 2/19; 13/19	Resection: 3/19 (2 surgeries and 1 combined) Shunt: 3/19
Stastna	− (12;11.5–12)	− (6.5; 4–14.2)	64/73; 3/73; 61/73	Resection: 6/73 SRS: 1/73 VP shunt: 4/73 Cystic puncture plus SRS: 1/73
Mezey et al	12.5(-;12–18)	12.4(-;7.8–21.5)	81/103; 60/103; 21/103	Resection:17/103 Shunt: 13/103 Ventriculostomy: 2/103
Hasegawa et al	− (12;9–13)	− (6.7;2–28.9)	168/203; 34/203; 134/203	Resection: 35/203 SRS: 6/203 VP shunt: 6/203
Umekawa et al	12(-;12–14)	5.3(-;4.1–6.8)	46/50	Resection: 2/50 Shunt: 2/50
Series excluding patients with prior surgery				
Van de Langenberg	12.6 (-; 12.5–13)	− (8.8; 6.1–17.7)	29/33; 7/33; 22/33	Resection: 5/33 (combined) SRS: 2/33 s Shunt: 2/33 (6 and 12 months after)
Milligan et al	− (12; 12–14)	− (9.4; 5.3–19.1)	18/22; 0/22; 18/22	Resection: 2/22 (median 32 (14–90) after) Shunt: 2/22
Bailo et al	− (13;11–15)	6.0 (5.6; 2.5–14.9) 25–30 mm vs > 30	54/59	Resection: 1/59 (48 months after) Shunt: 10/59
Huo et al	− (12.5;12–13.5)	− (4.06;3.5–6.99)	18/19	Resection: 1/19 Shunt: 3/19
Ogino et al	− (12.5; 10.5–22)	− (7.4;5–20)	Actuarial rates (not numbers)	MS:7/170 SRS:1/170 Shunt:8/170 after a median 7.2 (1.3–32.7)
Pikis et al	Median 12	Median 8.7	590/627; 299/627; 291/627	Resection:18/627 SRS and rhizotomy:1/627 SRS: 6/627 Cyst aspiration:1/627 Shunt: 7/627

### Exclusion criteria

We excluded studies written in languages other than English, duplicate studies from the same author or institution, and studies reporting fractionated stereotactic radiotherapy outcomes[15]. Those reports identified in the initial search strategy that included combined outcomes with either SRS or fractionated stereotactic radiotherapy were excluded if outcomes specific to the SRS cohort could not be segregated.

### Outcome measures

The primary outcome for this analysis was the radiographic local tumor control rate, however, the definition of tumor control varied considerably amongst publications. As an example, Chung et al.[12] considered “tumor regression” if the post-treatment volume was less than 100% of the pre-SRS volume, “stable disease” if the post-treatment volume was within 100–110% of the pre-SRS volume, and “disease progression” if the post-treatment volume was > 110% of the pre-SRS volume. In the largest study by Pikis et al.[28], local failure was defined as an increase in the total VS volume of more than 20% at last follow-up, while decrease was defined as a reduction in tumor volume of more than 20% from baseline at the last radiological follow-up. Given the heterogeneity in the definition of local control across studies and to facilitate summary statistics, we defined radiological local control as stability or a decrease in tumor volume at last follow-up (regardless of the degree of tumor reduction). We also considered treatment failure in those patients with tumor enlargement. Further microsurgical interventions, surgical management of hydrocephalus, delivery of further SRS, cystic puncture etc., were noted for the adverse event analyses and not counted as a treatment failure (Table 2). Adverse clinical outcomes specific to CN toxicities were summarized separately.

Follow-up periods are illustrated in Table 1 and are heterogeneous.

### Establishment of evidence based guidelines

The present systematic review has been performed by a group of international experts from a wide range of disciplines. Evidence was gathered from the primary literature. Recommendations, which are further summed, were made on the basis of this evidence and were graded in terms of their strength.

## Results

### Indication for SRS

Upfront SRS was considered by most authors for the following situations: (1) elderly patients, (2) lack of disabling symptoms, (3) presence of serviceable hearing, (4) comorbidities precluding candidacy for surgery, (5) and no symptomatic mass effect[11, 15, 16, 18, 21, 28].

## Tumor control

### Tumor control (stability or decrease)

**Fig. 2:**
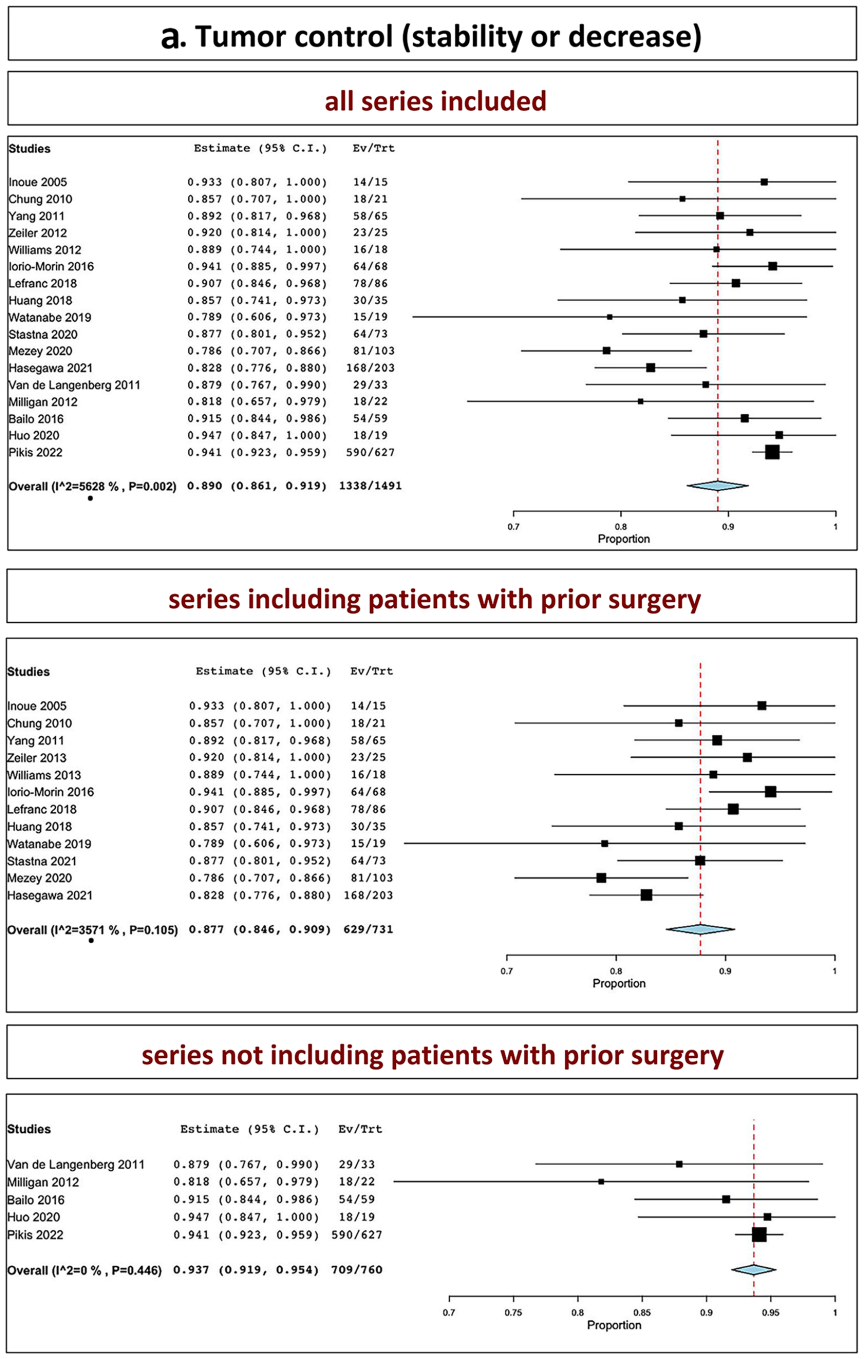

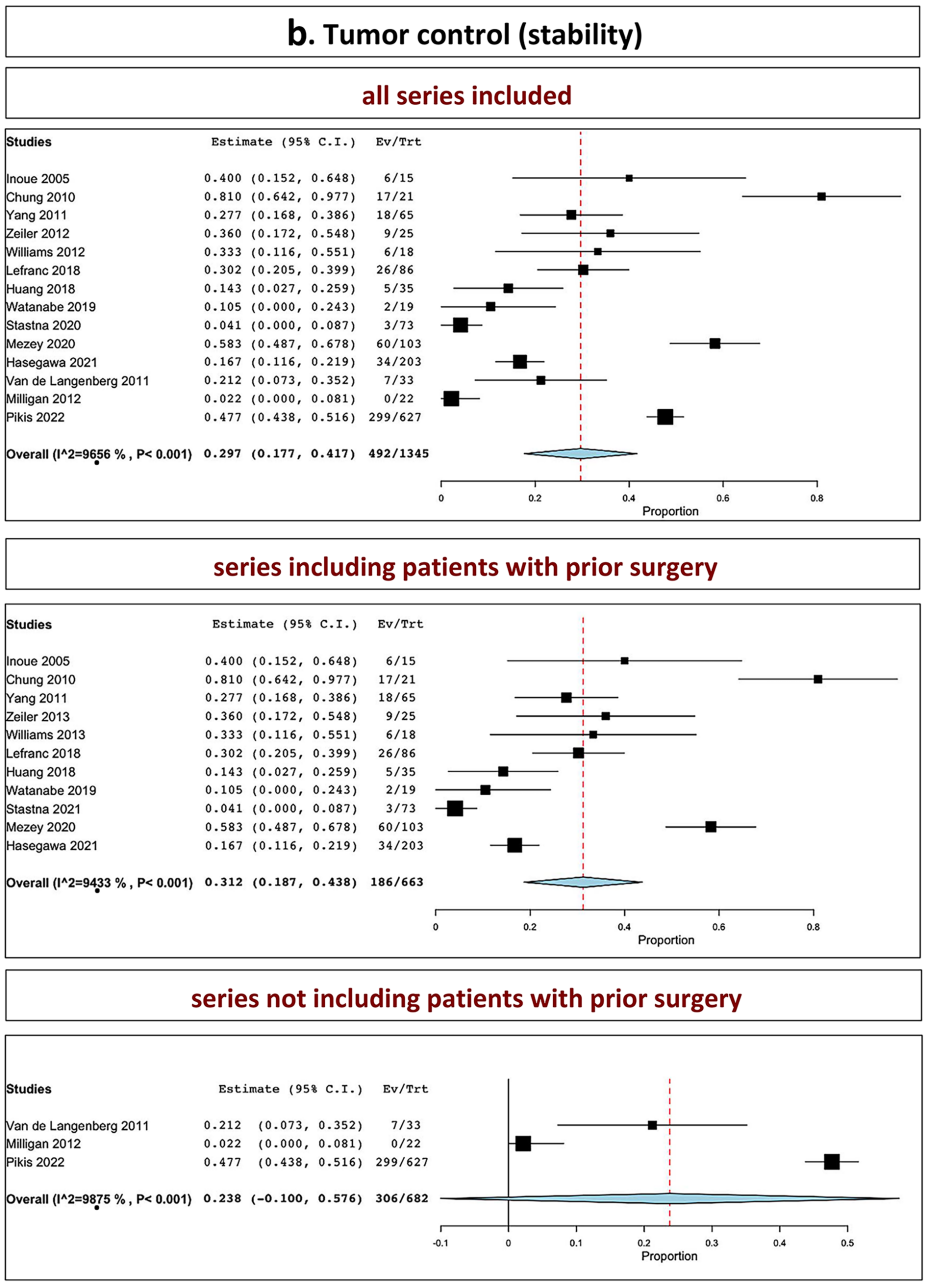

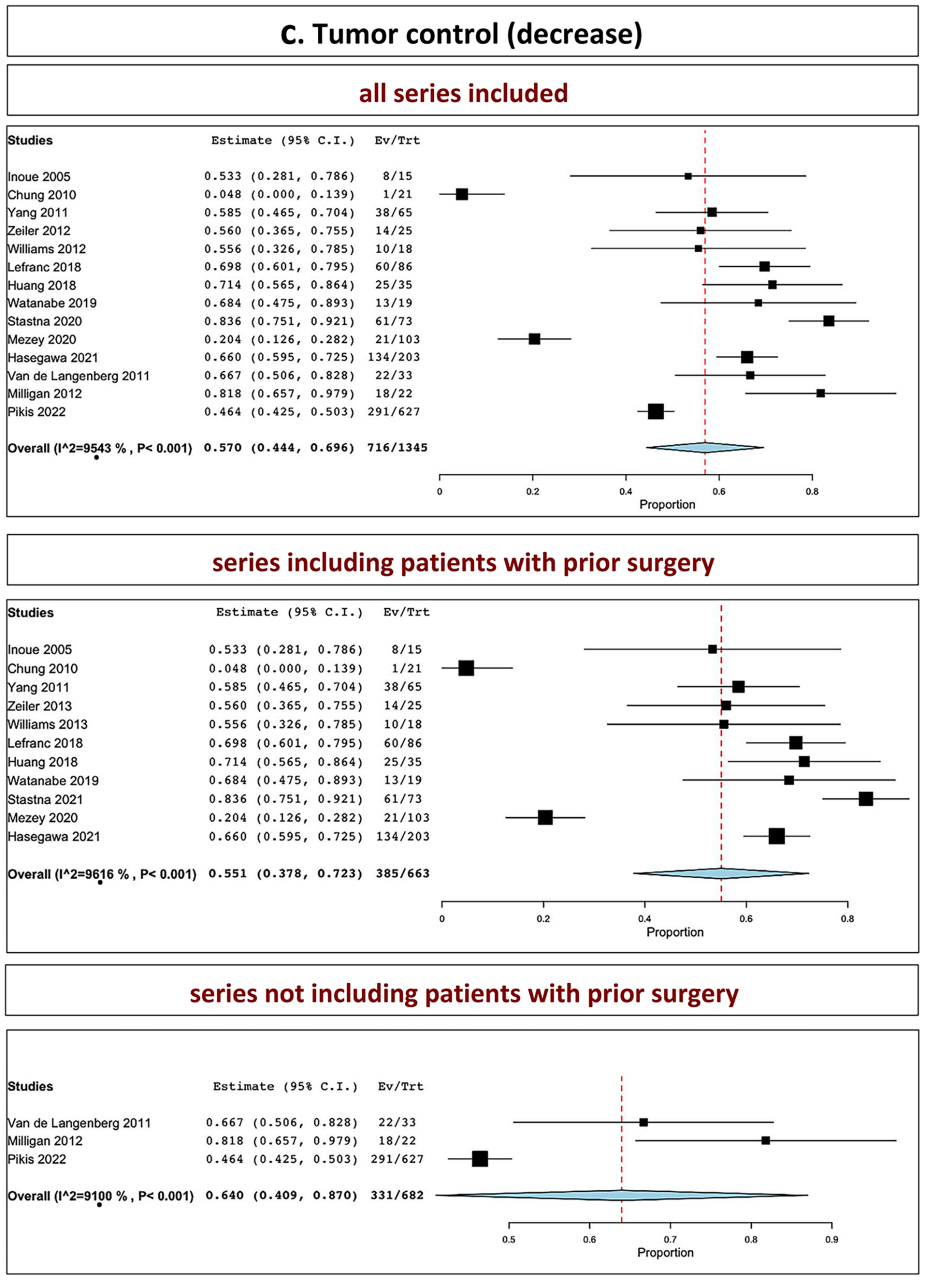
Tumor control: **A** stability and decrease included; **B** stability; **C** decrease (upper part: all series confounded; middle part: series including patients with prior surgery; lower part: series not including patients with prior surgery)

The overall tumor control in all series was 89.0% (range 86.1–91.9%, I^2^ = 56.28%, p heterogeneity = 0.002, *p* < 0.001; Fig. 2a, upper part). The overall tumor control in series including patients with prior surgery was 87.7% (range 84.6–90.9%, I^2^ = 35.71%, p heterogeneity = 0.1, *p* < 0.001; Fig. 2a, middle part). The overall tumor control in series not including patients with prior surgery was 93.7% (range 91.9–95.4%, I^2^ = 0%, p heterogeneity = 0.4, *p* < 0.001; Fig. 2a, lower part).

### Tumor stability

The overall tumor stability in all series was 29.7% (range, 17.7–41.7%, I^2^ = 96.56%, *p* heterogeneity < 0.001, *p* < 0.001; Fig. 2b, upper part). The overall tumor stability in series including patients with prior surgery was 31.2% (range 18.7–43.8%, I^2^ = 94.33%, p heterogeneity < 0.001, *p* < 0.001; Fig. 2b, middle part). The overall tumor stability in series not including patients with prior surgery was 23.8% (I^2^ = 98.75%, p heterogeneity < 0.001, *p* = 0.16; Fig. 2b, lower part).

### Tumor reduction

The overall tumor reduction in all series was observed in 57.0% (range 44.4–69.6%, I^2^ = 95.43%, *p* heterogeneity < 0.001, *p* < 0.001; Fig. 2c, upper part). The overall tumor reduction in series including patients with prior surgery was 55.1% (range 37.8–72.3%, I^2^ = 96.16%, p heterogeneity < 0.001, *p* < 0.001; Fig. 2c, middle part). The overall tumor reduction in series not including patients with prior surgery was 64.0% (40.9–87%, I^2^ = 91.0%, p heterogeneity < 0.001, *p* < 0.001; Fig. 2c, lower part).

### Transient-tumor-expansion (pseudoprogression)

TTE was inconsistently reported. Specifically, only 3/19[24] of the included studies described this outcome. One series reported a crude risk of 41% in 26 patients treated with a median time-to-onset of 8 months (range, 6–13)[17]. Regarding dosimetric predictors, in the series of Chung et al.[12] there was a significant correlation between the T2 signal ratio between tumor and brainstem and the duration of tumor swelling.

## Post-SRS procedures

### Salvage resection

**Fig. 3:**
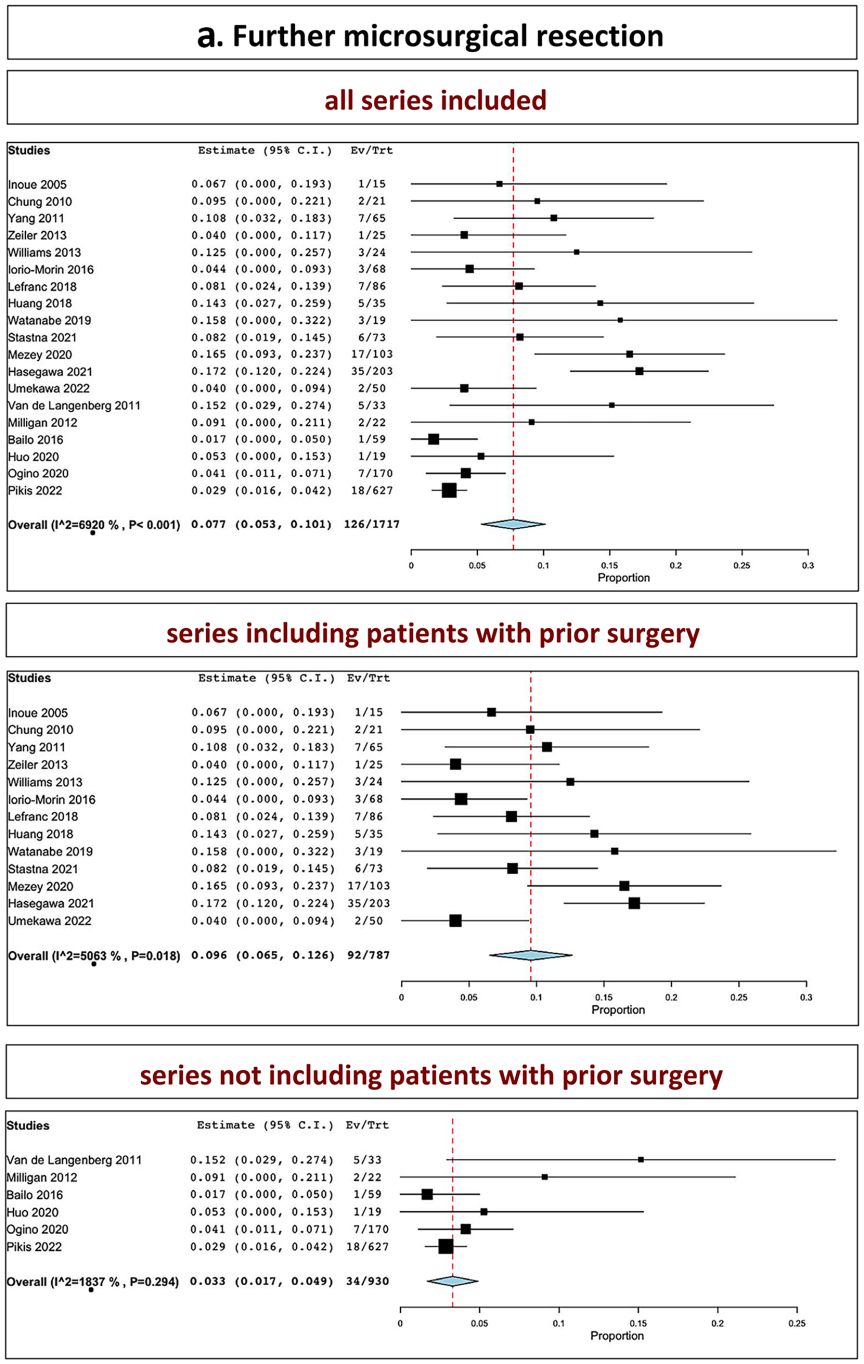

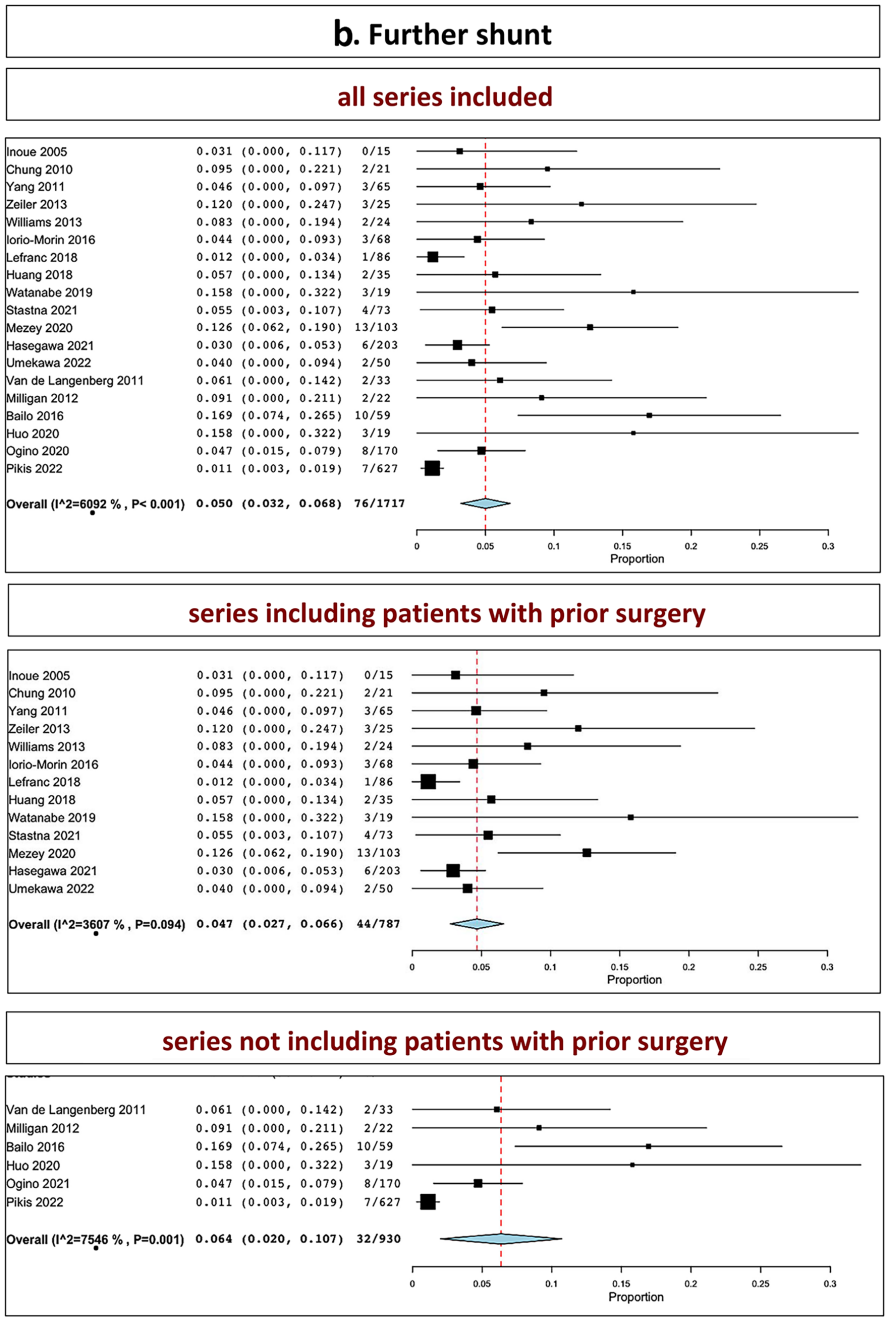

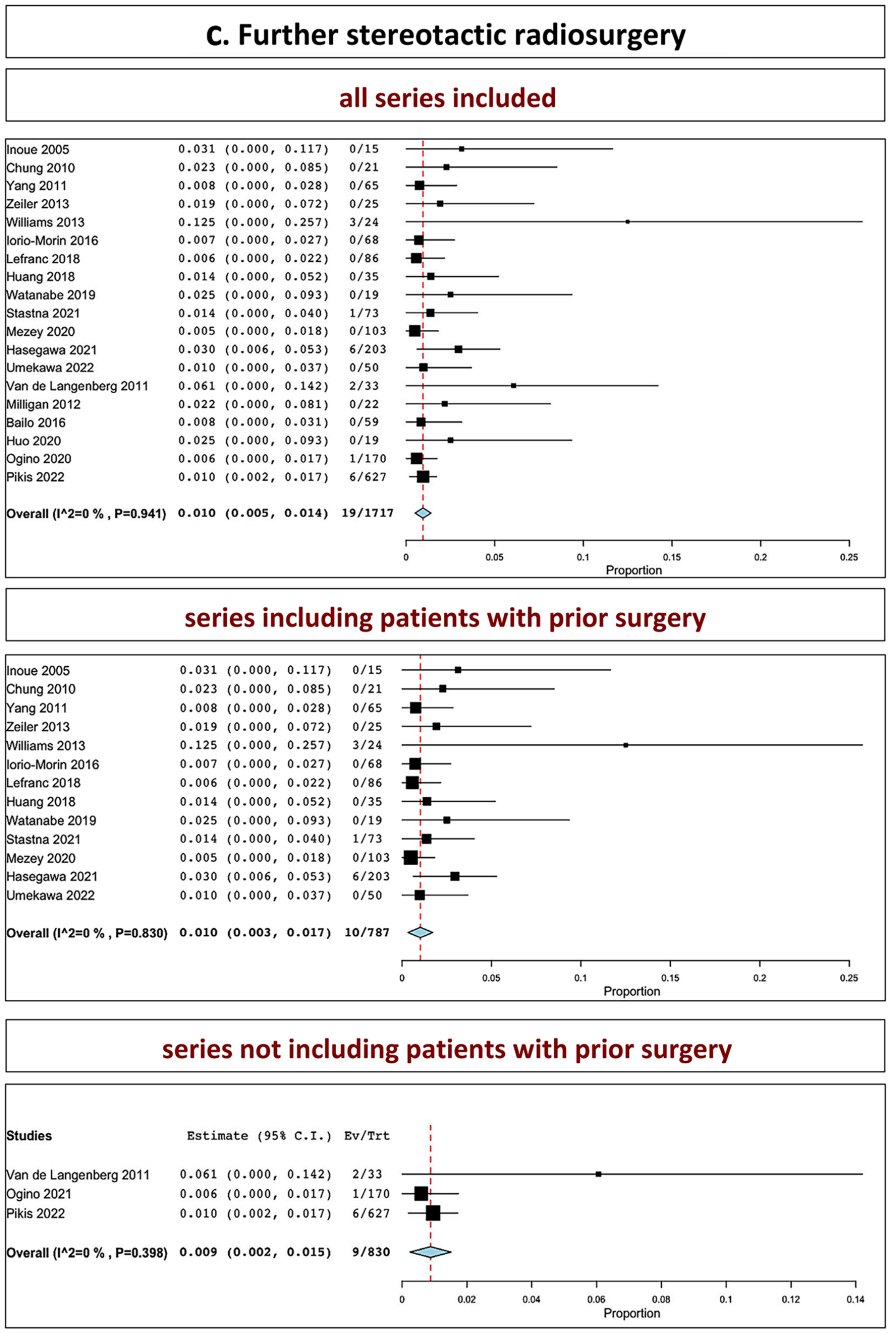
Further surgical intervention: **A** microsurgery; **B** shunt; **C** SRS (upper part: all series confounded; middle part: series including patients with prior surgery; lower part: series not including patients with prior surgery)

The overall rate of further microsurgical resection in all series was 7.7% (range 5.3–10.1%, I^2^ = 69.2%, *p* heterogeneity < 0.001, *p* < 0.001; Fig. 3, a, upper part). The overall rate of further microsurgical resection in series including patients with prior surgery was 9.6% (range 6.5–12.6%, I^2^ = 50.63%, *p* heterogeneity = 0.01, *p* < 0.001; Fig. 3, a, middle part). The overall rate of further microsurgical resection in series not including patients with prior surgery was 3.3% (range 1.7–4.9%, I^2^ = 18.37%, *p* heterogeneity = 0.29, *p* < 0.001; Fig. 3, a, lower part).

### Post-SRS shunting for hydrocephalus

The overall rate of need for shunt in all series was 5.0% (range, 3.2–6.8%, I^2^ = 60.92%, *p* heterogeneity < 0.001, *p* < 0.001; Fig. 3, b, upper part). The overall rate of further shunt placement in series including patients with prior surgery was 4.7% (range 2.7–6.6%, I^2^ = 36.07%, *p* heterogeneity = 0.09, *p* < 0.001; Fig. 3, b, middle part). The overall rate of further shunt placement in series not including patients with prior surgery was 6.4% (range 2–10.7%, I^2^ = 75.46%, *p* heterogeneity = 0.001, *p* < 0.001; Fig. 3, b, lower part).

### Further salvage SRS

The overall rate of salvage SRS in all series was 1.0% (range, 0.5–1.4%, I^2^ = 0%, *p* heterogeneity = 0.16, *p* = 0.941; Fig. 3, c, upper part). The overall rate of further salvage SRS in series including patients with prior surgery was 2.6% (I^2^ = 41.68%, *p* heterogeneity = 0.18; Fig. 3, c, middle part). The overall rate of further salvage SRS in series not including patients with prior surgery was 1.0% (range 0.3–1.7%, I^2^ = 0%, *p* heterogeneity = 0.83, *p* = 0.004; Fig. 3, c, lower part).

## Cranial nerve toxicities and hearing preservation

**Fig. 4:**
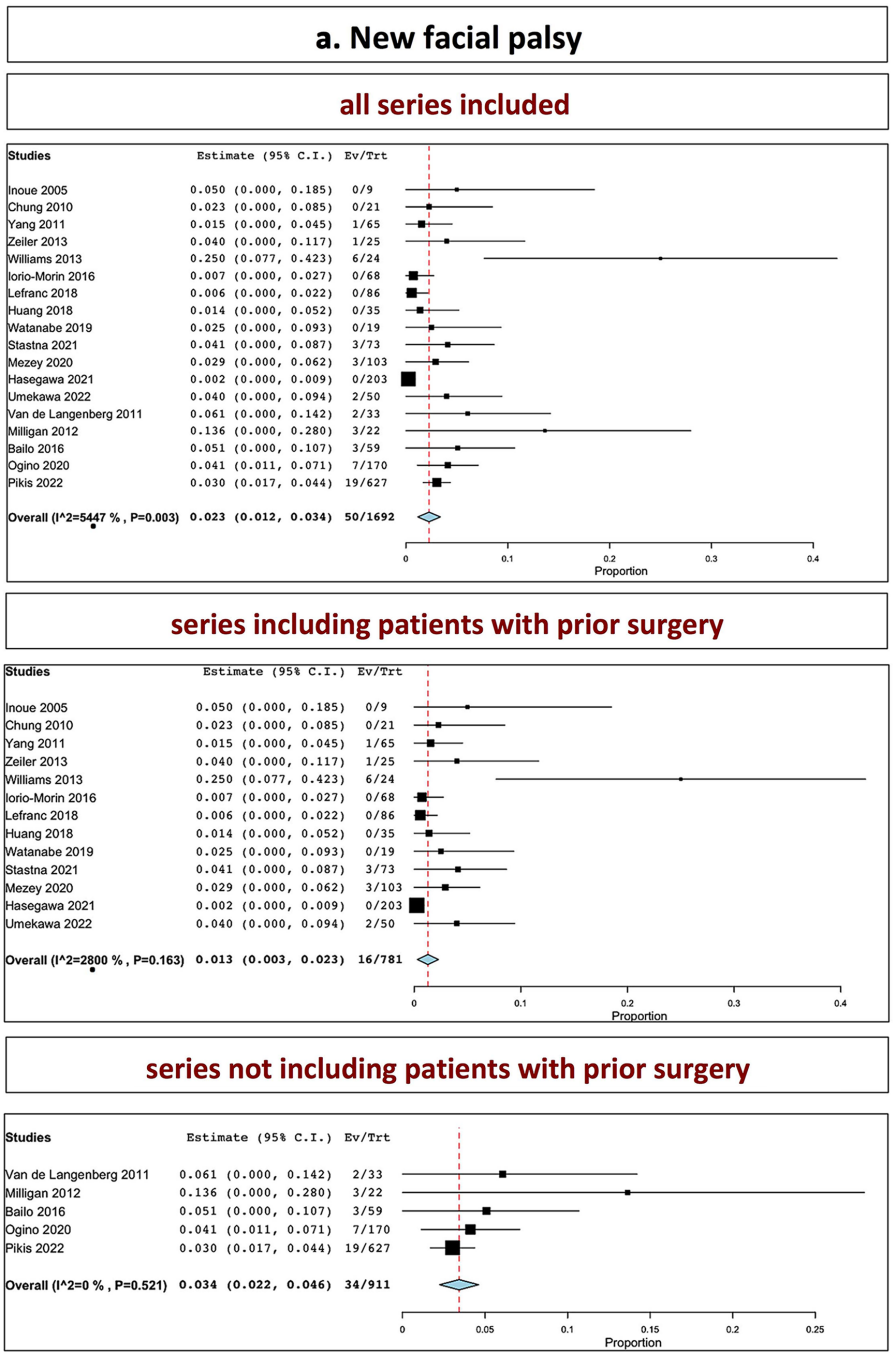

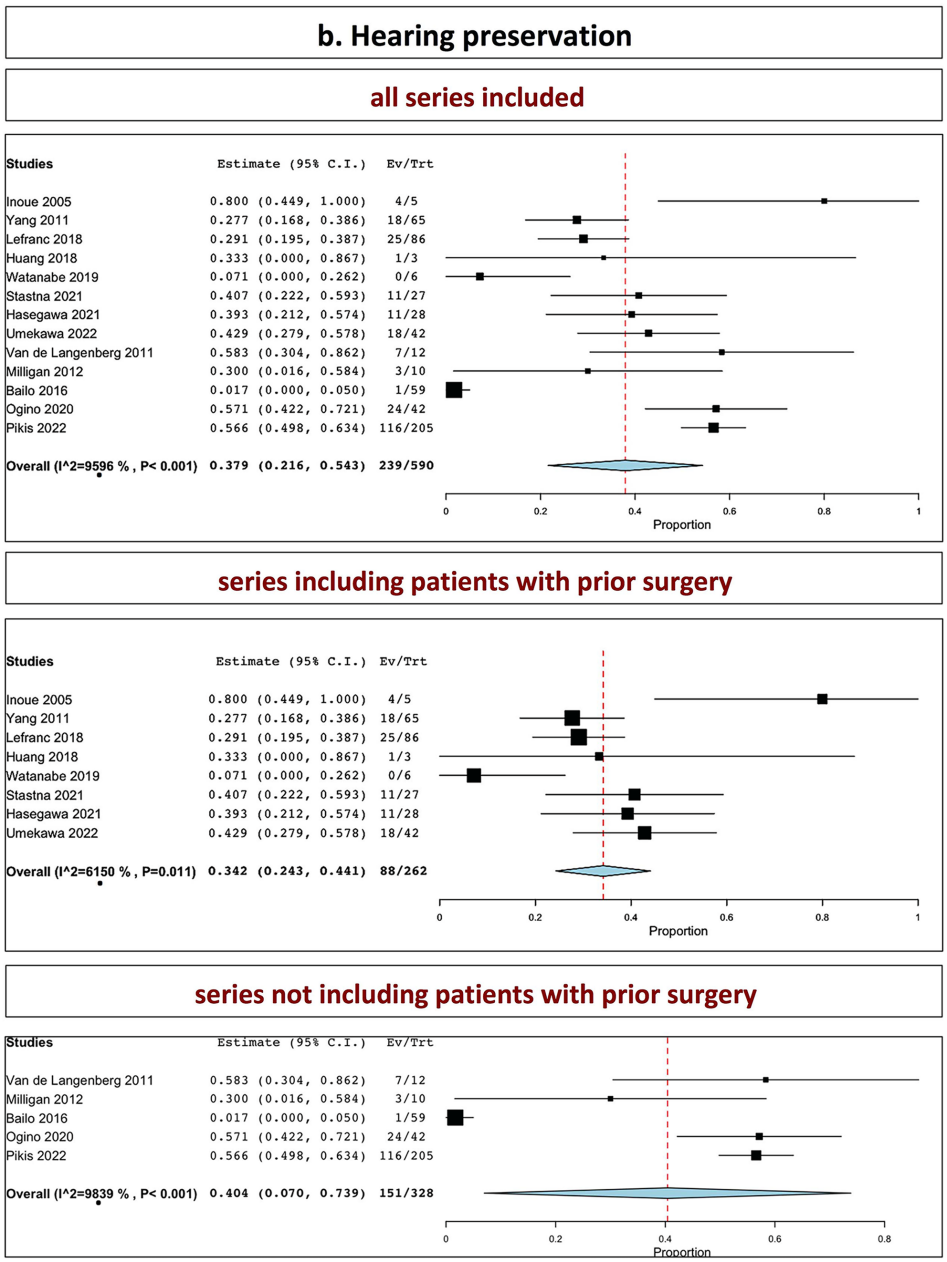
Relevant clinical outcomes: **A** new facial palsy; **B** hearing preservation rates (upper part: all series confounded; middle part: series including patients with prior surgery; lower part: series not including patients with prior surgery)

The overall rate of facial palsy in all series was 2.3% (range 1.2–3.4%, I^2^ = 54.47%, *p* heterogeneity = 0.003, *p* < 0.001; Fig. 4, a, upper part). The overall rate of new-onset facial palsy in series including patients with prior surgery was 1.3% (range 0.3–2.3%, I^2^ = 28.00%, *p* heterogeneity = 0.16, *p* = 0.01; Fig. 4, a, middle part). The overall rate of facial palsy in series not including patients with prior surgery was 3.4% (range 2.2–4.6%, I^2^ = 0%, *p* heterogeneity = 0.52, *p* < 0.001; Fig. 4, a, lower part).

The overall rate of hearing preservation in all series was 37.9% (range 21.6–54.3%, I^2^ = 95.96%, *p* heterogeneity < 0.001, *p* < 0.001; Fig. 4, b, upper part). The overall rate of hearing preservation in series including patients with prior surgery was 34.2% (range 24.3–44.1%, I^2^ = 61.5%, *p* heterogeneity = 0.01, *p* < 0.001; Fig. 4, b, middle part). The overall rate of hearing preservation in series not including patients with prior surgery was 40.4% (7.0–73.9%, I^2^ = 98.39%, *p* heterogeneity < 0.001, *p* = 0.06; Fig. 4, b, lower part).Tables 2, 3 and 4 summarizes the outcomes.

**Table 3:** Clinical outcome (worsening or new relevant deficits)

	Worsened facial palsy (preexisting)	New facial palsy /hemifacial spasm	Hearing preservation	Disturbance in balance	Other CN new deficit
Series including patients with prior surgery					
Inoue et al	0/9	0/9	4/5 up to 13 years	–	0/20
Chung et al	0/21	0/21	–	5/21	1 case of malignant transformation 1 case of cerebellar infarction
Yang et al	–	1/65 HB II at 6 months	18/22	–	4/65 sensory dysfunction
Zeiler et al	–	1/25 worsening hemifacial spasm	–	7/28 (temporary)	2/25 sensory dysfunction
Williams et al	2/24	6/24 worsening of preexisting	–	–	3/11 worsened sensory dysfunction
Iorio-Morin et al	–	0/68	–	8/68	4/68 sensory dysfunction
Lefranc et al	–	0/86	25/38	–	–
Huang et al	0/7	0/28	1/3	–	–
Watanabe et al	–	0/7	0/6	–	2/19 transient trigeminal neuropathy
Stastna	–	3/73 (stated as worsened of preexistent or new)	11/27	–	6/73 facial paresthesias 5/73 trigeminal neuralgia
Mezey et al	3/103	3/103 facial palsy 5/103 hemifacial spasm	–	12/103	6/103 facial paresthesias 3/103 trigeminal neuralgia
Hasegawa et al	–	0/203	11/28	–	11/203 trigeminal neuralgia 2 tumor-related deaths (one malignant transformation)
Umekawa et al	–	2/50	18/42	0/50	3/50
Series excluding patients with prior surgery					
Van de Langenberg et al	–	2/33 transient facial palsy (HB II)	7/12	1/33	3/33 facial paresthesias
Milligan et al	–	3/22 facial palsy 1/22 hemifacial spasm	3/10	–	3/22 trigeminal neuralgia
Bailo et al	–	3/59	1/59 transient worsening at 6 months	5/16	4/59 new/worsened trigeminal deficit
Huo et al	–	–	–	–	–
Ogino et al	–	7/170	24/42	10/170	15/170
Pikis et al	–	19/627	116/205	9/81	48/627

**Table 4:** Overall outcome (summary): tumor control, further intervention and cranial nerves

	All series together	P value	Series including patients with prior surgery	P value	Series not including patients with prior surgery	P value
Tumor control						
Tumor control (stability or decrease)	**89%** (86.1–91.9%)	< 0.001	**87.7%** (84.6–90.9%)	< 0.001	**93.7%** (91.9–95.4%)	< 0.001
Tumor stability	**29.7%** (17.7–41.7%)	< 0.001	**31.2%** (18.7–43.8%)	< 0.001	**23.8%**	0.16
Tumor reduction	**57%** (44.4–69.6%)	< 0.001	**55.1%** (37.8–72.3%)	< 0.001	**64%** (40.9–87%)	< 0.001
Further intervention						
Further microsurgery	**7.7%** (5.3–10.1%)	< 0.001	**9.6%** (6.5–12.6%)	< 0.001	**3.3%** (1.7–4.9%)	< 0.001
Further shunt	**5%** (3.2–6.8%)	< 0.001	**4.7%** (2.7–6.6%)	< 0.001	**6.4%** (2–10.7%)	< 0.001
Further SRS	**10%** (0.5–1.4%)	< 0.001	**1%** (0.03–1.7)	0.004	**0.9%** (0.2–1.5%)	0.007
Cranial nerve outcomes						
Facial nerve palsy	**2.3%** (1.2–3.4%)	< 0.001	**1.3%** (0.3–2.3%)	0.01	**3.4%** (2.2–4.6%)	< 0.001
Hearing preservation	**37.9%** (21.6–54.3%)	< 0.001	**34.2%** (24.3–44.1%)	< 0.001	**40.4%** (7.0–73.9%)	0.01

*numbers in bold correspond to overall rates

## Discussion

Our systematic review suggests that single fraction SRS could be used for VS ≥ 2.5 cm in maximum diameter, and/or Koos Grade IV, as either the primary treatment modality or for post-operative residual/recurrent tumor. However, we acknowledge that the Koos grade IV tumor definition varies significantly across studies and the minority (6 studies out of 19) of the published literature was specific to upfront treatment.

The overall probability of tumor control (both stability and decrease in volume) and tumor reduction in all series versus those series without prior surgery were 89% versus 93.7%, and 57% versus 64%, respectively. Several of the included series in this meta-analyses identified individual parameters associated with local failure. More specifically, Hasegawa et al. [13] suggested that a high-risk group for lower tumor control included patients with middle cerebellar peduncle compression of ≥ 9.8 mm and ≤ 48 years of age. Tumor control was also higher when prescribing a marginal dose of greater than 12 Gy as compared with less than 12 Gy [19] and for those smaller volumes [18]. Previous microsurgery, tumor volumes exceeding 10 mL, Koos grade IV [8], tumor volume more than 15 mL [14] and progression of residual disease preceding SRS [21] were also factors resulting in lower local control rates. These findings informed the ISRS recommendations as summarized in Table 5.

**Table 5:** Summary and recommendations

Summary (level C evidence)	
Tumor control	
Lower	Previous resection
	Volumes exceeding 10 mL
	Large Koos IV
	Progression of residual VS prior to SRS
	Middle cerebellar peduncle compression
Higher	Marginal dose of at least 12 Gy
	Smaller volumes
Facial nerve preservation (better)	Volume less than 10 mL
	Non-cystic VS
	Marginal dose lower than 13 Gy
Hearing preservation (better)	Age < 60 years
	Gardner-Robertson 1 at treatment
	Cochlear dose less than 4 Gy
Recommendations	
Ideal candidates: Patients • Without disabling symptoms, • With serviceable hearing, • With comorbidities that make resection riskier • Those who wish to avoid a resection, • With no symptomatic mass effect	
Marginal dose prescription: Between 11 and 13 Gy	

The rates of salvage microsurgical resection, need for shunt, and additional SRS in all series versus those series with no prior surgery were 9.6% vs 3.3%, and 4.7% vs 6.4% and 1% vs 0.9%, respectively. The rates of facial palsy and hearing preservation in all series versus those series with no prior surgery were 2.3% vs 3.4% and 37.9% vs 40.4%, respectively. Preservation of the facial nerve function was associated with smaller tumor volumes (less than 10 mL) and lower margin dose (≤ 13 Gy) [19]. Deterioration of facial nerve function was associated with a prescription dose of ≥ 13 Gy and early TTE [28]. Hearing preservation was higher in patients with good pre-therapeutic levels of hearing (Gardner Robertson class 1), younger age, and a dose of less than 4 Gy to the cochlea/modiolus (the mean dose/point dose of less than 4 Gy to the cochlea/modiolus being already reported in the literature during the past 15 years and in the overall context of hearing preservation after SRS for VS [29, 30]). Cranial nerve complication rates were suggested in few of reviewed studies to be greater in those VS with cystic components vs solid [11]. Trigeminal neuropathy was rare and usually transient.

A particular entity that would deserve further analysis, although limited data exist on such topic, is related to previously irradiated large partially cystic VSs that will potentially develop symptomatic mass effect from fluid-dynamic cyst enlargement [31, 32], without definite neoplastic growth of the solid part. In such patients, microsurgical exploration for cyst fenestration/drainage without the need for further resection of the already treated tumor cells can be a valuable option, in the absence of tumor growth of the solid part. Such surgical option could reveal much safer and with less morbidity, in the absence of planned subtotal resection of the solid, non-growing part.

In the present review, the overall need for shunt for large VSs treated with upfront SRS in series without prior surgery was 6.4%, which is much higher as compared to smaller tumors. Previous series have suggested the need for shunt after SRS after a median time of 15.5 months (range 1.8–37.8) [33]. Hydrocephalus after radiosurgery may thus co-occur with a temporary tumor volume change after radiation and there is a crucial need for careful ongoing clinical and imaging follow-up [33]. Other authors suggested that large tumor size, ring enhancement patterns and high protein level of CSF should be carefully observed during follow-up course [34]. Thus, using programmable/adjustable MR-compatible ventriculo-peritoneal shunts in time might prevent devastating consequences due to increased intracranial pressure and a risk of sudden neurological decline.

Of the 1723 cases in this meta-analysis, four tumor-related deaths were observed. Two were secondary to developed malignant transformation, which accounts for 0.12% of the sample. This low risk is consistent with the literature including a recent meta-analysis [35]. The other two deaths were related to a refractory VS which relapsed 78 months from the time of SRS and the second due to tumor-related subarachnoid hemorrhage (Hasegawa et al.^14^). Moreover, TTE was inconsistently reported and should be better detailed by further studies on the same topic. Such TTE might be, in some cases, accompanied by acute and subacute radiation effects, which are in vast majority of cases transient [36].

With respect to fractionated radiation, there are as yet limited data with regards to the use of hypofractionnated SRS for large VS [13, 37]. However, there are 6 non-randomized trials [38–43] comparing single fraction SRS with fractionated stereotactic radiotherapy (FSRT). There has yet to be significant differences in 5-year tumor control rates between the two techniques to make any firm recommendations. A recent systematic review compared SRS versus FSRT for tumor control in VSs [44]. The authors suggested that the progression-free survival rates were 92–100% for both treatment options, while the risk of facial and trigeminal nerve deterioration was less for patients treated with SRS [44]. It has been also acknowledged that there is a lack of high-quality studies comparing radiation therapy alternatives for patients with VSs [44]. We would still support fractionated stereotactic radiotherapy for large VS given the established practice as a standard of care, and experience in other benign tumors with favorable control rates [45, 46]. However, there is a need for a randomized or prospectively controlled trial comparing single fraction SRS and FSRT in VS, especially in the context of clarifying if functional outcome would be better with single fraction SRS.

The main limitation of the present meta-analysis was the inability to reliably separate outcomes between upfront vs salvage cohorts, and this added complexity to this analyses. We acknowledge that those studies including patients with prior surgery also included cases with upfront SRS, which can contribute to added bias. The definition of large tumors was also extremely heterogeneous. In particular, for those treated in the post-operative residual or recurrent setting. A limited number of series included “staged-volume” SRS strategies, which might have also influenced local control. The same applies to the cystic tumors, which influence the overall results in terms of local control, and in some series they account for as high as 58% of the included cases [24]. However, the results for cystic tumors have not been separately reported in individual series, although it is now well acknowledged that they respond best to SRS as compared to the solid ones [31]. Additionally, there was a lack of uniformity with regards to the follow-up periods, to which ads variations depending on studies to the long-term and even the short-term follow up. Tumor diameters were inconsistently reported. There was also a lack of reported actuarial outcomes, which are different from the crude rates reported in the studies. There were also several different nuances concerning further neurosurgical interventions, considered as adverse events and not counted as treatment failures. Particularly, the surgical management of hydrocephalus was heterogeneous, including ventriculo-peritoneal shunt, ventriculostomy, Ommaya placement or further cyst puncture and timing of further SRS and additional surgical interventions were also extremely variable, as well as for further surgical interventions.

### Recommendations

Ideal candidates for SRS in patients with a VSs of a maximum diameter ≥ 2.5 cm and/or classified as Koos Grade IV are those without symptomatic mass effect, without disabling symptoms, with pre-SRS serviceable hearing, and with comorbidities that make resection more risky or those who wish to avoid a resection (class C evidence). Based on the analyzed data, we conclude that local tumor control is optimal when prescribing a marginal dose between 11 and 13 Gy (class C evidence). Lower rates of tumor control were associated with prior surgical resection, volumes exceeding 10 mL, large Koos grade IV, progression of residual VS prior to SRS and middle cerebellar peduncle compression (class C evidence). Better facial nerve preservation was observed when treating tumor volumes less than 10 mL, non-cystic VS, and when thw marginal dose lower is than 13 Gy (class C evidence). Better hearing preservation rates were associated with younger patients (age less than 60 years), better initial hearing level (Gardner-Robertson 1) and a cochlear dose of less than 4 Gy (class C evidence). The ISRS recommendations are summarized in Table 5.

## Conclusion

Although large VS are considered a classical indication for microsurgical resection, upfront single fraction SRS might be useful in select patients (class C evidence). When analyzing data from those series with no prior surgery vs those with prior surgery, higher rates of tumor control, further tumor reduction, lower rates of further intervention (microsurgical resection, shunt, SRS), higher rates of *“*de novo*”* facial palsy (although overall low) and higher hearing preservation rates were observed (class C evidence).

## Data Availability

The datasets generated during and/or analysed during the current study are available from the corresponding author on reasonable request. They are however described in details in tables and figures.
